# Cobalt protoporphyrin promotes human keratinocyte migration under hyperglycemic conditions

**DOI:** 10.1186/s10020-022-00499-0

**Published:** 2022-06-23

**Authors:** Peng-Hsiang Fang, Ying-Ying Lai, Chih-Ling Chen, Hsin-Yu Wang, Ya-Ning Chang, Yung-Chang Lin, Yu-Ting Yan, Cheng-Hung Lai, Bill Cheng

**Affiliations:** 1 Department of Veterinary Medicine, National Chung-Hsing University, No.145, Xing Da Road, 402 Taichung, Taiwan; 2 Bachelor Program of Biotechnology, National Chung-Hsing University, Taichung, Taiwan; 3 Graduate Institute of Biomedical Engineering, National Chung-Hsing University, No.145, Xing Da Road, 402 Taichung, Taiwan; 4 Institute of Biomedical Science, Academia Sinica, Taipei, Taiwan

**Keywords:** Cobalt protoporphyrin, Keratinocytes, Re-epithelialization, Diabetic Wound

## Abstract

**Background:**

Complete healing of diabetic wounds continues to be a clinically unmet need. Although robust therapies such as stem cell therapy and growth factor treatment are clinically applied, these treatments are costly for most diabetic wound patients. Therefore, a cheaper alternative is needed. Cobalt protoporphyrin (CoPP) has recently been demonstrated to promote tissue regeneration. In this study, the therapeutic benefits of CoPP in diabetic wound healing were examined.

**Methods:**

An in vitro wound healing model that mimics re-epithelialization was established to examine the effect of CoPP on the migratory capability of human keratinocytes (HaCaT) in either normal glucose (NG) or high glucose (HG) media, as well as in the presence of either H_2_O_2_ or lipopolysaccharide (LPS). At the end of the migration assays, cells were collected and subjected to Western blotting analysis and immunostaining.

**Results:**

HaCaT were found to migrate significantly more slowly in the HG media compared to the NG media. CoPP treatment was found to enhance cell migration in HG media, but was found to decrease cell migration and proliferation when HaCaT were cultured in NG media. CoPP treatment induced high levels of expression of Nrf-2/HO-1 and FoxO1 in HaCaT cultured in either glucose concentration, although the FoxO1 expression was found to be significantly higher in HaCaT that underwent the migration assay in NG media compared to those in HG media. The higher level of FoxO1 expression seen in CoPP-treated HaCaT cultured in NG media resulted in upregulation of CCL20 and downregulation of TGFβ1. In contrast, HaCaT migrated in HG media were found to have high levels of expression of TGFβ1, and low levels of expression of CCL20. Interestingly, in the presence of H_2_O_2_, CoPP-pretreated HaCaT cultured in either NG or HG media had similar expression level of Nrf-2/HO-1 and FoxO1 to each other. Moreover, the anti-apoptotic effect of CoPP pretreatment was noticed in HaCaT cultured in either glucose concentration. Additionally, CoPP pretreatment was shown to promote tight junction formation in HaCaT suffering from LPS-induced damage.

**Conclusions:**

CoPP enhances cell migratory capacity under hyperglycemic conditions, and protects cells from oxidative and LPS-induced cellular damage in HG media containing either H_2_O_2_ or LPS.

**Supplementary Information:**

The online version contains supplementary material available at 10.1186/s10020-022-00499-0.

## Introduction

Diabetes is a classic example of a chronic disease that continues to be a financial burden to many countries’ healthcare systems. It has been estimated that in Taiwan, a country that has a high prevalence of diabetic mellitus (DM), ~ 15% of DM patients suffer from diabetic wounds (Tai et al. 2021). On average, these patients can spend 12.7 ± 11.8 days in hospitals (Cheng et al. 2019), which can incur a huge cost to most patients and their families, both financially and emotionally. To date, stem cell therapy is the most effective method of treating any diabetic wound, as treatment enhances re-epithelization and angiogenesis, and downregulates profound immune response (Carstens et al. 2021). However, the cost per treatment is enormous, especially when most of the patients with diabetic wounds are characterized as low income (Tai et al. 2021). Accordingly, there is a growing need for non-stem cell related therapy that could be used as a cheaper alternative for patients that with less severe diabetic wounds (Li et al. 2021).

Developing small molecules that can induce the expression of heme oxygenase-1 (HO-1) is considered a promising therapy for wound healing and tissue regeneration. HO-1 is an enzyme that catalyzes the breakdown of heme into biliverdin, carbon monoxide (CO) and iron (Li et al. 2018). Both CO and biliverdin, and the final heme catabolic end-product bilirubin, are known to have strong antioxidant and anti-inflammatory activities, and the by-product iron has been demonstrated to participate in ferritin synthesis, in which ferritin has antiapoptotic activity (Maamoun et al. 2017). Thus, due to its ability to produce by-products that are beneficial to tissue regeneration, there has been a growing interest in developing small molecules that can promote tissue regeneration through the induction of HO-1 expression. One of the small molecules that have attracted attention is known as cobalt protoporphyrin (CoPP). To date, CoPP has been demonstrated to attenuate liver injury (Li et al. 2018), enhance the anti-tumour effect of bortezomib in adult T-cell leukaemia cells (Hamamura et al. 2007), as well as provide cardioprotection to the ischemia-reperfusion injured heart(Cheng et al. 2016).

CoPP is a synthetic derivative of protoporphyrin IX (PPIX), which is an organic compound that occurs naturally and is a precursor of heme in the human body (Hamamura et al. 2007). Structurally, PPIX contains a porphine core, a tetrapyrrole macrocycle with a marked aromatic character, with the two inner hydrogen atoms being replaced by a divalent metal cation (Hamamura et al. 2007). Thus, heme is a molecule that consists of PPIX complexed with an iron (II) (ferrous) cation Fe^2+^. Likewise, when PPIX is complexed with a cobaltous cation, Co^2+^, the molecule is known as CoPP (Li et al. 2018). In fact, CoPP shares a structure similar to vitamin B_12_, in which the Co^2+^ occupies the center of a corrin in the latter, as opposed to a porphine in the former (Osman et al. 2021). Clinically, vitamin B_12_ has been validated for its importance in blood maturation(Green 2017), and CoPP has been demonstrated to increase endogenous granulocyte colony-stimulating factor and enhance the mobilization of hemopoietic stem cells (Szade et al. 2019). Furthermore, vitamin B_12_ has also been shown to promote healthy skin development (Rembe et al. 2018). Thus, it is reasonable to presume that CoPP will likely to have a positive effect on re-epithelization of a diabetic wound. In the present study, the therapeutic effect of CoPP in promoting re-epithelization under different conditions was demonstrated in in vitro wound healing assay using a live-cell imaging system.

## Materials and methods

### Chemicals and antibodies

Cobalt protoporphyrin (Enzo Life Science, Cat# ALX-430-076-M025) was prepared in 0.1 M NaOH, in which the final stock concentration was 25 mg/mL. Hydrogen peroxide (Cat# H1009, Sigma-Aldrich) was freshly prepared according to the required concentration in the culture media. Lipopolysaccharide (LPS, Cat# L2018, Sigma-Aldrich) was dissolved in autoclaved ultrapure water, and prepared as a 5 mg/mL stock solution. Anti-human HO-1 antibody (Cat# E-AB-18,231, Elabscience) and anti-human CCL20 antibody (E-AB-65,380, Elabscience) was purchased from Blossom Biotechnologies, Taiwan. Anti-human β-actin antibodies (GTX109639), anti-human Nrf-2 antibody (GTX135165), anti-human FoxO1 antibody (GTX135251), anti-human TGFβ1 antibody (GTX130023), anti-human Akt (phosphorylated) antibody (GTX128414), anti-human Akt (internal control) antibody (GTX121937), and anti-human ZO-1 (GTX108592) antibody were purchased from GeneTex, Taiwan. Anti-human caspase 3 (cleaved) antibody (ARG57512), anti-human caspase 3 (pro) antibody (ARG65765), anti-human p38 MAPK antibody (ARG55258) and anti-human p38 MAPK (phosphorylated) antibody (ARG51850) antibody, and anti-human Bax antibody (ARG65612) were purchased from Arigo Biolaboratories, Taiwan. The working concentration for all the primary antibodies and secondary antibodies were 1:1000 and 1:10,0000 respectively.

### Cell culture

DMEM-low glucose (Cat# 11,885,084, Gibco) containing 10% foetal bovine serum (FBS, Cat# 10,437,028, Gibco) and 1% penicillin and streptomycin (P/S, Cat# 15,140,122, Gibco) were used for the culturing of human keratinocytes, HaCaT (Cat# 300,493, CLS) under normoglycemic conditions. For hyperglycemic conditions, HaCaT were cultured in DMEM-high glucose (Cat# 11,965,092, Gibco) containing 10% FBS and 1% P/S. Human skin fibroblasts, WS1 (Cat# CRL-1502, ATCC) were cultured in EMEM-low glucose (Cat# 30-2003, ATCC) containing 10% FBS and 1% P/S. To investigate the effect of high glucose to WS1, glucose (Cat# 49,163, Sigma-Aldrich) was added to the EMEM media to 25 mM.

### Cell viability

The numbers of viable cells were determined by using a haemocytometer (Merck, Cat# Z359629). For each sample, cells were trypsinized, centrifuged, and the pellet was diluted in an appropriate amount of PBS buffer. Subsequently the cells were stained with trypan blue (ThermoFisher Scientific, Cat# 15,250,061) to differentiate the non-viable from viable cells. The cell viability was determined with the formula below:

$$\% viable\,cells = [1.00{\text{ - }}\,(Number\,of\,blue\,cells{\text{ }} \div Number\,of\,total\,cells)]{\text{ }} \times 100$$

### In vitro wound healing assay

Ibidi stage top incubation system (Cat# 10,720, Ibidi) was used for the live cell imaging of HaCaT migration. The cells were first seeded in a 2-well culture-insert (Cat# 80,209, Ibidi) in low glucose media overnight. The next day, the culture-insert was removed and HaCaT were live-imaged in either low or high glucose media with or without 10 µM CoPP. For the analysis of HaCaT migration in the presence of either H_2_O_2_ or LPS, cells were pretreated with CoPP for > 12 h before adding either 300 µM H_2_O_2_ or 1 µg/mL LPS to the media, followed by live-cell imaging.

### Dodecyl sulfate polyacrylamide electrophoresis (SDS-PAGE) and western blotting

SDS polyacrylamide gels (4–12%, Cat# HC2040, ThermoFisher Scientific) were prepared according to the manufacturer’s protocol using Mini-PROTEAN Tetra Handcast Systems (Cat# 1658000FC, Bio Rad). Samples were electrophorized at 100 V at room temperature until the dye front had reached the bottom of the gel. Subsequently, the samples were transferred onto polyvinylidene fluoride (PVDF) membrane (0.45 μm, Cat# IPVH00010, Merck Millipore). After blocking overnight at 4 °C, the membrane was exposed to primary antibodies at 4 °C with shaking overnight one more time, and then washed with 0.1% TBST before applying secondary antibodies for 1 h at room temperature with shaking. After washing with 0.1% TBST, the signals were detected with ECL-plus reagent (Cat# WBKLS0500, Merck Millipore) and imaged with Odyssey Fc Imaging System (LI-COR Biosciences). The intensity of each band was quantified with Image Studio™ Software 5.x (LI-COR Biosciences). All the experiments were repeated three times and the representative western blots were presented.

### Immunofluorescence staining

The cells were first seeded onto glass slides and incubated at 37 °C overnight. The next day, the cells were exposed to 10 µM CoPP in either DMEM-low glucose or DMEM-high glucose for 12 h at 37 °C. The slides were then washed with warm PBS, and the CoPP-pretreated cells were exposed to 1 ug/mL LPS in either DMEM-low glucose or DMEM-high glucose at 37 °C overnight. After washing in PBS, the cells were fixed with 4% paraformaldehyde at 4 °C overnight. The fixed cells were then washed with cold PBS, and incubated with anti-ZO-1 antibodies at 4 °C with shaking overnight. PBST (0.1%) was used to wash away any unbound anti-ZO-1 antibodies, and the cells were then incubated with FITC-conjugated secondary antibodies in the dark at room temperature for one hour. The cells were washed with cold 0.1% PBST, and imaged with a fluorescence microscope (Nexcope, China). The relative fluorescence intensity was measured with a digital scanner (Pannoramic 250 FALSH II), and result was analyzed with Pannoramic Viewer.

### Statistical analysis

Students t test (for two samples, assuming equal variance) was used to compare statistical significance. A P value of less than 0.05 was considered significant.

## Results

### Human keratinocytes exhibited a slower migration rate under hyperglycemic conditions

**Fig. 1:**
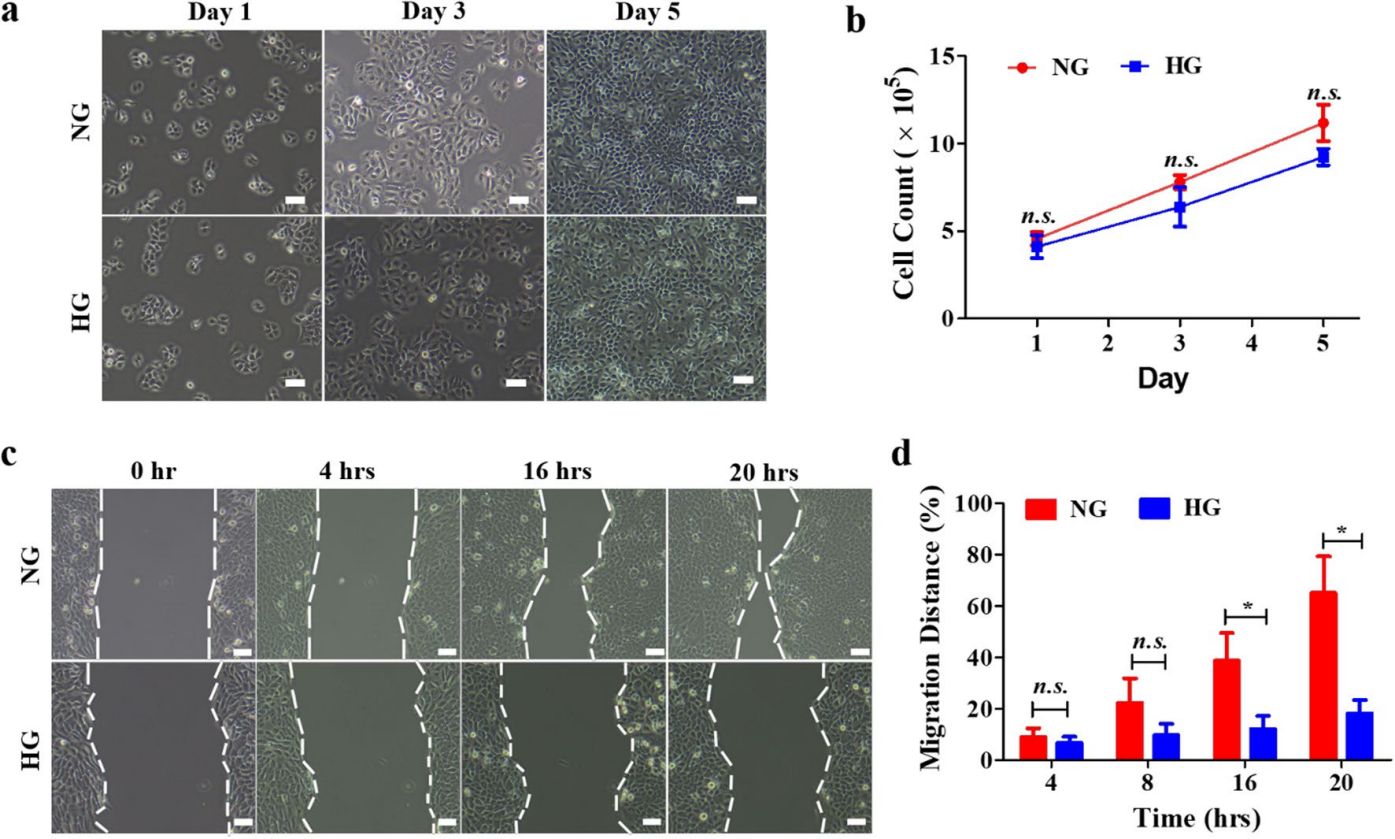
Proliferation and migration of HaCaT under normoglycemia and hyperglycemia conditions. Human keratinocytes, HaCaT were cultured in normal glucose (NG) or high glucose (HG) media and **a** images were taken at Day 1, 3, 5 after seeding (scale bar, 100 μm). **b** Viable cell counts of either culture throughout the 5 days of culturing were determined. **c** Time lapse images showing the migrated distance of HaCaT cultured in either NG or HG media (scale bar, 100 μm), and **d** the statistical analysis of the migrated distance of the two groups. *, *P* < 0.05; *n.s.* not significant

Recent in vivo studies have indicated hyperglycemia had a strong influence on the therapeutic outcome of applied therapeutics (Gnyawali et al. 2020; Tan et al. 2021). Thus, prior to evaluating the therapeutic actions of CoPP, a reliable and physiologically relevant in vitro model was established (Fig. 1). It is well established that the blood glucose level of a healthy person is in the range of 3.5–5.5 mM, and that a person with blood glucose level greater than 11.0 mM at 2 h after meals is likely to suffer from internal organ damage (Güemes et al. 2016). Accordingly, human keratinocytes (HaCaT) were cultured in commercially available media that contained either 5.5 mM (normal glucose, NG) or 25 mM (high glucose, HG) glucose. There was no significant difference in cell morphology and viable cell counts between the HaCaT that were cultured in either NG or HG media (Fig. 1a, b). On the contrary, in the cell migration assay that was designed to recapitulate the re-epithelization event in wound healing (Rodrigues Neves et al. 2019), a significant difference was noticed in the cell migration distance between the HaCaT cultured in NG media and those cultured in HG media (Fig. 1c, d). The time lapse experiment revealed the HaCaT had overall faster migration rate when cultured under normoglycemic conditions, and that significant differences were noticed after 16 h of cell migration. Like the analysis of cell proliferation, no abnormality was noticed in the cell morphology as the cells migrated towards the center in either NG or HG media.

### Long-term exposure to CoPP decreased cell proliferation in NG media, but not in HG media

**Fig. 2:**
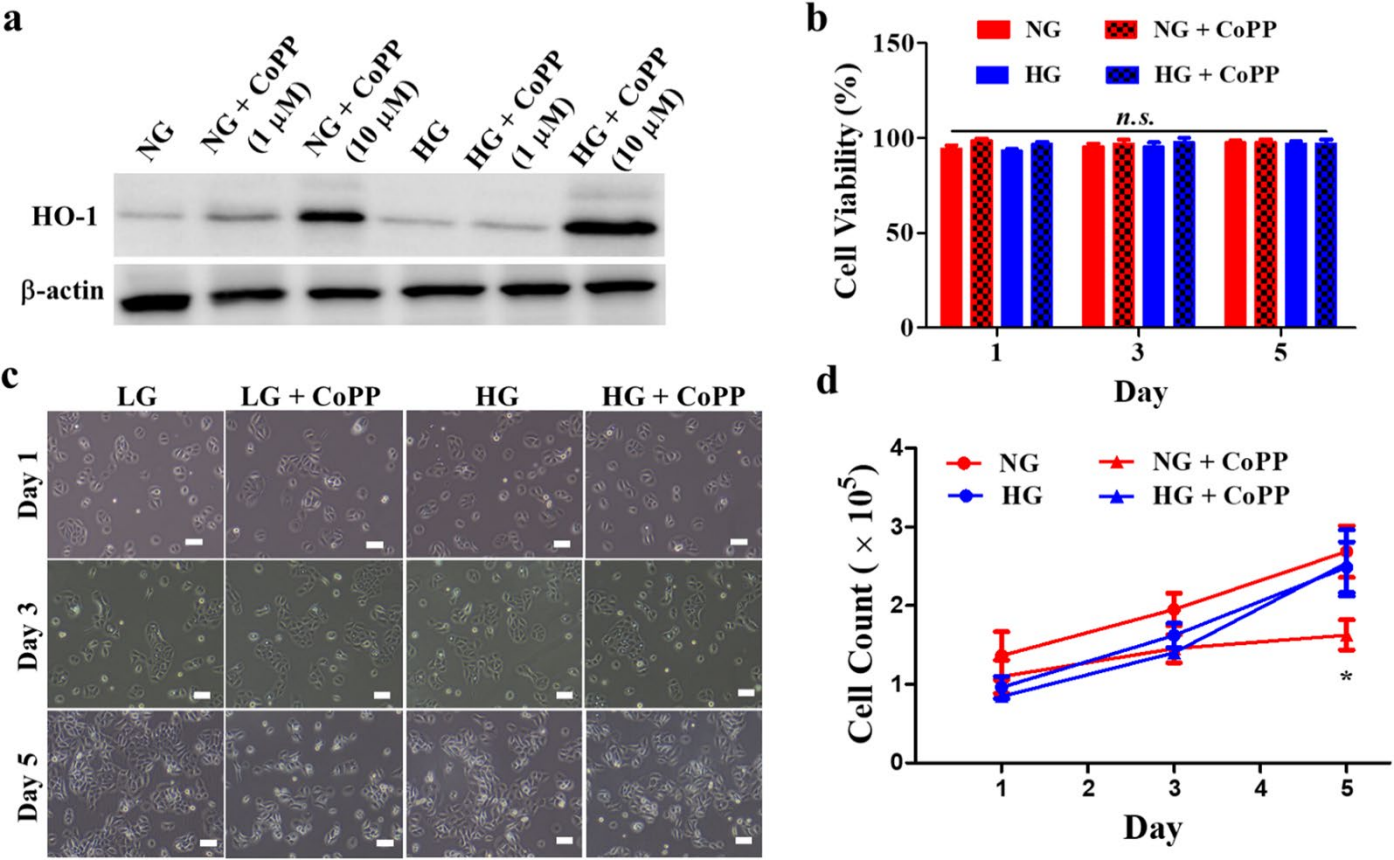
Proliferation of HaCaT culturedin the presence of CoPP. **a** Western blotting analysis of HO-1 in HaCaT cultured in either NG or HG media, after exposing to 1 µM or 10 µM of CoPP. **b** The effect of 10 µM of CoPP on the viability of HaCaT cultured in either NG or HG media. **c** The effect of 10 µM of on the morphology of HaCaT throughout the 5 days of exposure in either NG or HG media (scale bar, 100 μm). **d** Statistical analysis of numbers of HaCaT cultured in either CoPP-containing NG or HG media. *P < 0.05; *n.s.* not significant

Studies have revealed that 10 µM of CoPP could significantly enhance tissue regeneration in diseases such as myocardial infarction and ischemic renal injury(Cheng et al. 2016; Li et al. 2018). To demonstrate that the same concentration of CoPP could also induce strong expression of HO-1 in human keratinocytes, HaCaT cultured in either NG or HG media were exposed to either 1 µM or 10 µM of CoPP (Fig. 2a). Exposure to 1 µM of CoPP induced very little expression of HO-1 in HaCaT cultured in either medium. Contrastingly, strong expression of HO-1 was detected in HaCaT, after exposing the cells to 10 µM of CoPP in both media. Hence, the results indicated that the expression levels of CoPP-induced HO-1 were not affected by glucose concentration, and that strong HO-1 expressions were detected in both media that contained 10 µM of CoPP. Accordingly, 10 µM of CoPP were used for all subsequent experiments in the present study.

Since cobalt is a heavy metal, to demonstrate CoPP does not induce cell toxicity, viability of HaCaT were evaluated after exposing the cells to 10 µM of CoPP for 5 days. Compared to non-treated HaCaT in either NG or HG media, the addition of 10 µM of CoPP did not induce significant cell death (Fig. 2b) nor was there any abnormality in the cell morphology during the 5-day period of culturing (Fig. 2c). However, a significant difference was noticed in the numbers of cell counts at day 5 (Fig. 2d), where the numbers of CoPP-treated HaCaT cultured in NG media were significantly lowered compared to other treatment groups, including the CoPP-treated HaCaT cultured in HG media.

### CoPP enhanced human keratinocyte migration in HG media, but not in NG media

**Fig. 3:**
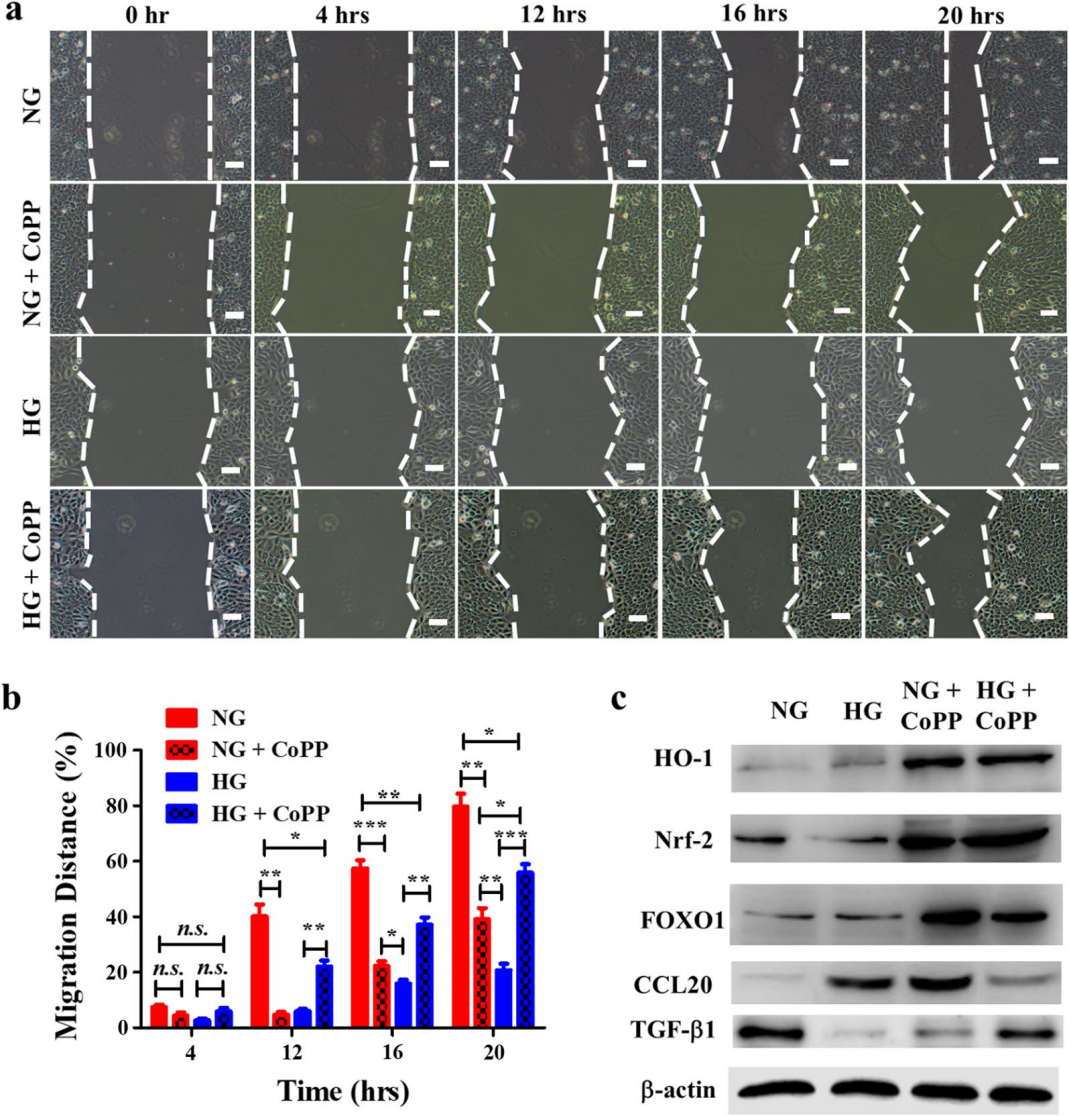
The influence of CoPP on the motility of HaCaT in vitro wound healing assay. **a** Time lapse images showing the migrated distance of HaCaT after exposing to CoPP containing NG or HG media (scale bar, 100 μm), **b**) and the statistical analysis. *P < 0.05; **P < 0.01; ***P < 0.001. **c** Western blotting analysis of cell lysate harvested from the HaCaT that had been subjected to the wound healing assay

Since CoPP were demonstrated to enhance and lower HaCaT proliferation after 5 days of culturing in HG and NG media, respectively (Fig. 2d), it was decided to see whether the molecules had any effect on the cell migration in a wound healing assay. HaCaT were cultured in a 2-well insert overnight, followed by exposure to 10 µM CoPP for at least 12 h before the insert was removed for the subsequent wound healing assay (Additional file 1: Fig. S1). The time-lapse results revealed that when comparing HaCaT cultured in non-CoPP treated NG media, HaCaT that received CoPP-treated NG media displayed slower cell migration (Fig. 3a). Contrastingly, profound enhancement in cell migration was seen in the HaCaT cultured in HG media after the addition of CoPP. Compared to the non-CoPP treated HaCaT cultured in HG media, those that received CoPP treatment migrated at a significantly faster rate in the same media (Fig. 3b) throughout the 20 h of monitoring. Notably, although CoPP had significantly enhanced HaCaT migration in HG media, the wound closure rate was still significantly slower compared to the non-CoPP treated HaCaT cultured in NG media. Interestingly, the migration rate of CoPP-treated HaCaT in NG media was significantly faster than the non-CoPP treated HaCaT in HG media, and yet the former was also significantly slower than the CoPP-treated HaCaT in HG media.

Similar to the response seen in HaCaT, the human skin fibroblasts, WS1, also migrated significantly slower in HG media (Additional file 1: Fig. S2a). Interestingly, the CoPP treatment did not enhanced the migration of human skin fibroblast, WS1 in either NG or HG media; instead, the treatment significantly decreased WS1 migration in both NG and HG media (Additional file 1: Fig. S2b).

### CoPP induced strong expressions of FoxO1 in human keratinocytes

A previous study demonstrated that the HO-1 level in human osteoarthritis (OA) cartilages from type 2 diabetes mellitus (T2DM) after CoPP treatment was significantly lower than those from non-T2DM (Vaamonde-Garcia et al. 2017). Given the contrasting effect of CoPP seen in HaCaT cultured in NG media versus those cultured in HG media, it was decided to investigated whether the difference in glucose concentration had any effect on the protein expressions of HO-1 and its upstream regulator, nuclear factor erythroid 2-related factor 2 (Nrf2) after CoPP treatment (Dong et al. 2021). At the end of the migration assays, cells from each treatment group were collected and subjected to Western blotting analysis (Fig. 3c). In the absence of CoPP, HaCaT cultured in either NG or HG media showed limited expression of HO-1, whereas the HO-1 expression in CoPP-treated HaCaT was significantly elevated when cultured in either medium (Additional file 1: Fig. S3). Similarly, strong expression of Nrf2 was detected in CoPP-treated HaCaT cultured in either NG or HG medium (Fig. 3c). In the absence of CoPP, the expression of Nrf-2 in HaCaT cultured in NG was significantly higher than HaCaT cultured in HG media (Additional file 1: Fig. S2). Collectively, the CoPP treatment was showed to affect cell migration depending on the glucose concentrations that the cells were cultured in. Moreover, the treatment had significantly increased the Nrf2 and HO-1 expressions regardless what glucose level that the cells were cultured in.

In addition to Nrf2, the expression of transcription factor forkhead box O1 (FoxO1) is induced upon CoPP exposure (Fig. 3 C). Upon CoPP induction, FoxO1 could also bind to the promoter of HO-1, and facilitate the transcription of the protein (Kang et al. 2014). Moreover, FoxO1 could to promote keratinocyte proliferation and migration under oxidative stress (Liu et al. 2013). The expressions of FoxO1 were upregulated in CoPP-treated HaCaT cultured in either NG or HG media compared to those that did not receive CoPP. However, the level of FoxO1 in CoPP-treated HaCaT was significantly higher when being cultured in NG media compared to those cultured in HG media (Additional file 1: Fig. S2). Similarly, glucose concentration also had a significant impact on the expressions of CCL20 and TGF-β1 in HaCaT (Fig. 3c). CCL20, also known as macrophage inflammatory protein-3 (MIP3A), is an inflammatory cytokine that is known to hinder keratinocyte migration during wound healing (Zhang et al. 2015). In contrast, TGF-β1 enhances the migration rate of keratinocytes during wound healing, thus fast-tracking the re-epithelization process (Zhang et al. 2015). In the absence of CoPP, minimal CCL20 expressions were detected in HaCaT cultured in NG; whereas strong CCL2 expression was seen in HaCaT cultured in HG media (Fig. 3c). Unexpectedly, CoPP treatment significantly increased the CCL20 expression in HaCaT cultured in NG media, and yet downregulated the level of the inflammatory cytokines in HaCaT cultured in HG media (Additional file 1: Fig. S3). Conversely, TGF-β1 expression was downregulated in CoPP-treated HaCaT cultured in NG media, but was significantly upregulated in CoPP-treated HaCaT cultured in HG media (Fig. 3c, Additional file 1: Fig. S3).

CoPP treatments resulted in contrasting effect in the protein expressions seen in WS1 that migrated in either NG or HG media (Additional file 1: Fig. S4). Strong HO-1 expressions were noticed in the cells that cultured in either medium after CoPP treatment. Interestingly, without CoPP treatment, WS1 displayed significantly stronger expressions of FoxO1 in HG media compared to those that underwent cell migrations in NG media, and that strong expressions of FoxO1 were noticed in CoPP treated WS1 that had undergone cell migration in either NG or HG media. Strong TGF-β1 expressions were only noticed in the cells that were in NG media without CoPP treatment, whereas all other treatment groups displayed significantly stronger CCL20 expressions.

### CoPP enhanced human keratinocyte migration in the presence of H_2_O_2_

It is widely established that the presence reactive oxygen species (ROS) is a predominant cause that hindered re-epithelization during the healing of diabetic wounds. To demonstrate that CoPP could promote cell survival from ROS damage, the migration of HaCaT, with or without CoPP pretreatment, cultured in either NG or HG media were examined after the addition of 300 µM H_2_O_2_. HaCaT cultured in either NG or HG media were first exposed to 10 µM CoPP for > 12 h, followed by washing in PBS, they were then were incubated in fresh media containing 300 µM H_2_O_2_. Cell death was clearly seen in both NG and HG media after 24 h of exposure to H_2_O_2_ (Additional file 1: Fig. S5a). The surviving cells cultured in HG media mostly displayed cell shrinkage features, in contrast to the well-adhered cell morphology seen in the surviving HaCaT cultured in NG media. The CoPP pretreatment significantly enhanced cell viability in the presence of H_2_O_2_ in both culture media, and that the treatment resulted in higher numbers of viable cells in the NG media than in the HG media, although the difference was not significant (Additional file 1: Fig. S5b).

**Fig. 4:**
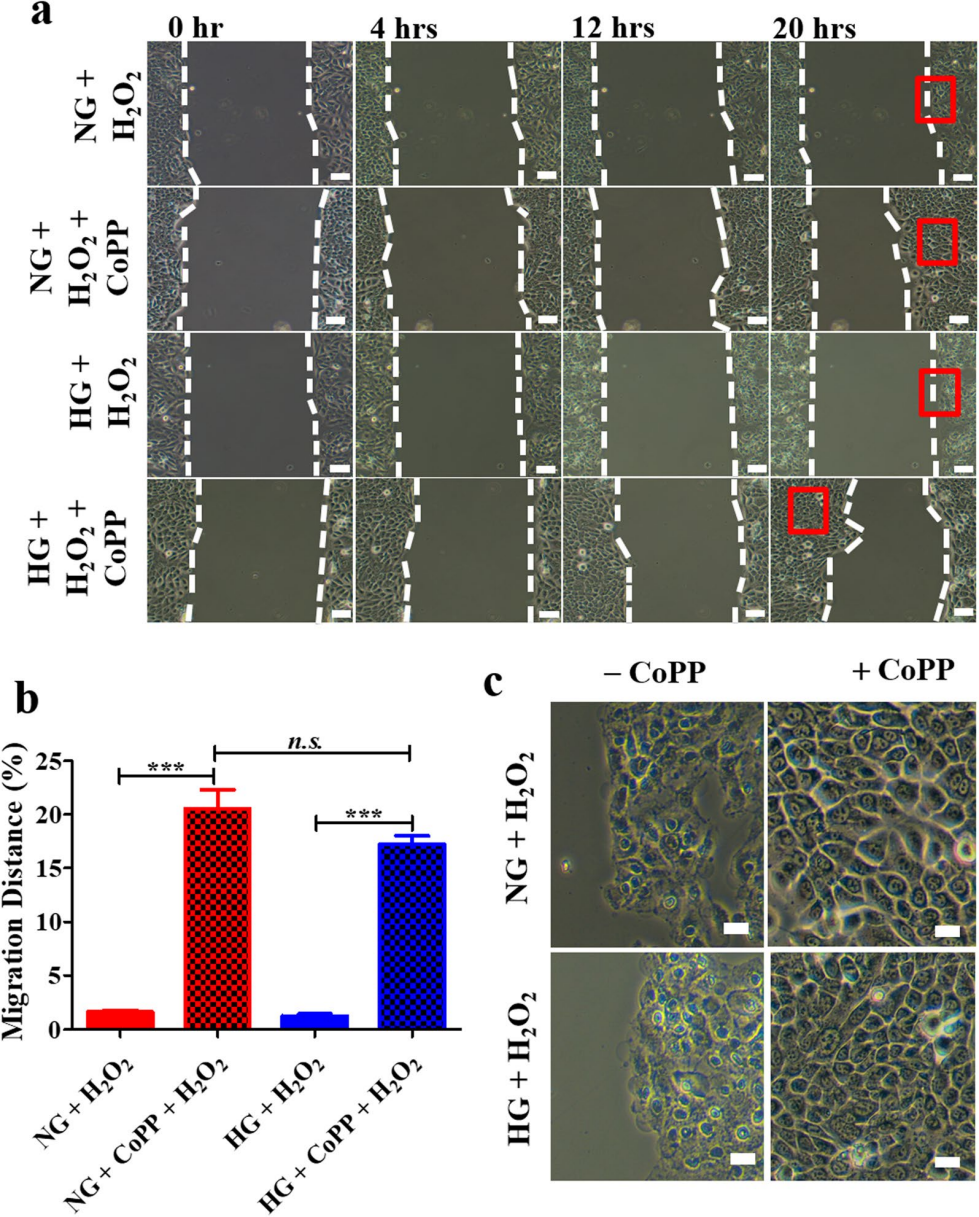
The protective effect of CoPP pretreatment in HaCaT after exposing to H_2_O_2_-containing media. **a** Time lapse images showing the migrated distance of CoPP-pretreated HaCaT after exposing to H_2_O_2_-containing media, **b** and the statistical analysis of the migrated distance of each sample at 20th hours of the migration assay. **c** The enlarged images of HaCaT after completed to the wound healing assay under different conditions (red box in (**a**)). Scale bar, 100 μm; ***P < 0.001; *n.s.* not significant

To examine whether CoPP pretreatment could also enhance cell migration in the presence of H_2_O_2_, a wound healing assay consisting of HaCaT cultured in either NG or HG media was exposed to 10 µM CoPP for at least 12 h, followed by exposure to culture media containing 300 µM H_2_O_2_ (Additional file 1: Fig. S6). The time lapse monitoring experiment revealed that cells that were pre-treated with CoPP displayed a faster migration rate in either medium when compared to those that did not receive the pretreatment (Fig. 4a), Although, when cultured in the presence of H_2_O_2_, CoPP-pretreated HaCaT displayed faster migration in NG media than in HG media, the difference between the migration distance in both treatment groups was not significant (Fig. 4b). Moreover, the HaCaT that had received CoPP pretreatment showed less apoptotic cell morphology in either medium when compared to the cells that did not receive the pretreatment (Fig. 4c). Collectively, although the response of HaCaT to CoPP treatment is influenced by glucose concentration, in the presence of H_2_O_2_, CoPP promoted cell survival and enhanced cell motility in the wound healing assay regardless of the glucose concentration in the media.

### CoPP pretreatment downregulated pro-apoptotic and upregulated anti-apoptotic gene expression in human keratinocytes that underwent cell migration in media containing H_2_O_2_

**Fig. 5:**
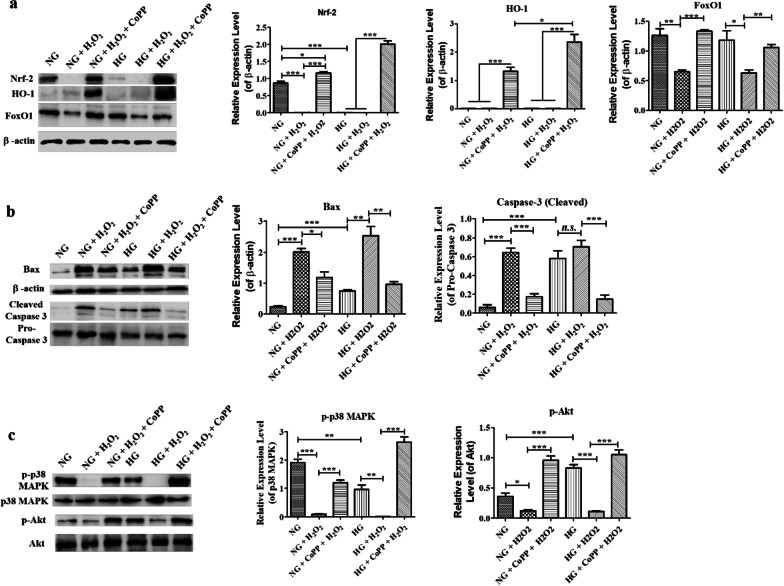
Western blotting analysis of pro-apoptotic and anti-apoptotic protein expressions in CoPP-pretreated HaCaT in H_2_O_2_-containing media. **a** The protein expression levels of Nrf-2, HO-1, and FoxO1. **b** The protein expression levels of Bax and cleaved caspase-3. **c** The protein levels of p-p38 MAPK and p-Akt. *P < 0.05; **P < 0.01; ***P < 0.001

To further understand the cytoprotective roles of CoPP in enhancing HaCaT motility in an in vitro wound healing assay consisting of 300 µM H_2_O_2_, cells were collected at the end of the assay and subjected to Western blotting analysis (Fig. 5). In the absence of CoPP, Nrf-2 expression was not detectable in HaCaT after they were cultured in either NG or HG media that contained 300 µM H_2_O_2_. Contrastingly, the pretreatment of CoPP elevated the Nrf-2 expression significantly in HaCaT cultured in either NG or HG media (Fig. 5a). Moreover, the CoPP pretreated HaCaT had significantly higher Nrf-2 expression than those that did not, even though the former was cultured in the presence of H_2_O_2_. As expected, CoPP pretreatment also resulted in a high level of HO-1 expression in HaCaT cultured in H_2_O_2_-containing NG or HG media (Fig. 5a). Interestingly, the pretreatment resulted in significantly higher expression of HO-1 in HaCaT cultured in H_2_O_2_-containing HG media than those cultured in H_2_O_2_-containing NG media (Fig. 5a), although the underlying reason is not clear. Likewise, the CoPP pretreatment restored the expressions of FoxO1 in HaCaT cultured in either H_2_O_2_ containing NG or HG media to the similar expression level seen in those cultured in either NG or HG media (Fig. 5a). Thus, the data suggests that CoPP could alleviate cellular damage from oxidative stress via inducing the expression of Nrf-2/HO-1 and FoxO1, regardless of the glucose concentration in the media.

Proteins such as Bax and caspase-3 that are known to induce cell apoptosis were notably downregulated in HaCaT that had received CoPP pretreatment (Fig. 5b**)**. Moreover, the addition of H_2_O_2_ significantly enhanced both Bax and cleaved caspase-3 (active form) in HaCaT cultured in either NG or HG media, and that expression was significantly downregulated in HaCaT that had received CoPP pretreatment **(**Fig. 5b). Therefore, the data indicated that CoPP-induced Nrf-2/HO-1 and FoxO1 could protect HaCaT from H_2_O_2_-induced cell apoptosis under both normoglycemic and hyperglycemic conditions.

In addition to downregulating the pro-apoptotic proteins, CoPP pretreatment significantly elevated the level of phosphorylated p38-mitogen-activated protein kinase (p-p38-MAPK) in HaCaT cultured in either H_2_O_2_-containing NG or HG media (Fig. 5c**)**. It is well-established that p-p38-MAPK is critical for promoting cell migration during re-epithelization, especially in the presence of ROS (Li et al. 2019). It was also noted that the expressions of p-p38-MAPK were downregulated in HaCaT cultured in HG media compared to those cultured in NG media, which further demonstrated that HaCaT migration was indeed inhibited under hyperglycemic conditions (Fig. 5c**)**. Without CoPP pretreatment, the expression level was further downregulated in both H_2_O_2_-containing NG and HG media. Similar results were also seen in the expression level of p-Akt (Fig. 5c), in which Akt is known to promote survival and growth in response to extracellular signals such as hyperglycemia-induced apoptosis (Li et al. 2019). In the absence of either CoPP pretreatment or H_2_O_2_, the expressions of p-Akt detected in HaCaT cultured in NG media were significantly lower than those cultured in HG, which indicated the hyperglycemic condition induced apoptotic stress (Fig. 5c); and that the HaCaT cultured in HG media had elevated the p-Akt expression in order to maintain cell survival. However, since the oxidative damage exerted by H_2_O_2_ was too strong, the overall p-Akt expression in HaCaT that were cultured in either H_2_O_2_-containing NG or HG media was significantly reduced (Fig. 5c). Nevertheless, the CoPP pretreatment resulted in stronger expression of p-Akt compared to the other two groups, regardless of whether the cells were cultured in H_2_O_2_-containing NG or HG media.

### CoPP enhanced the migration of keratinocytes in LPS-containing HG media

**Fig. 6:**
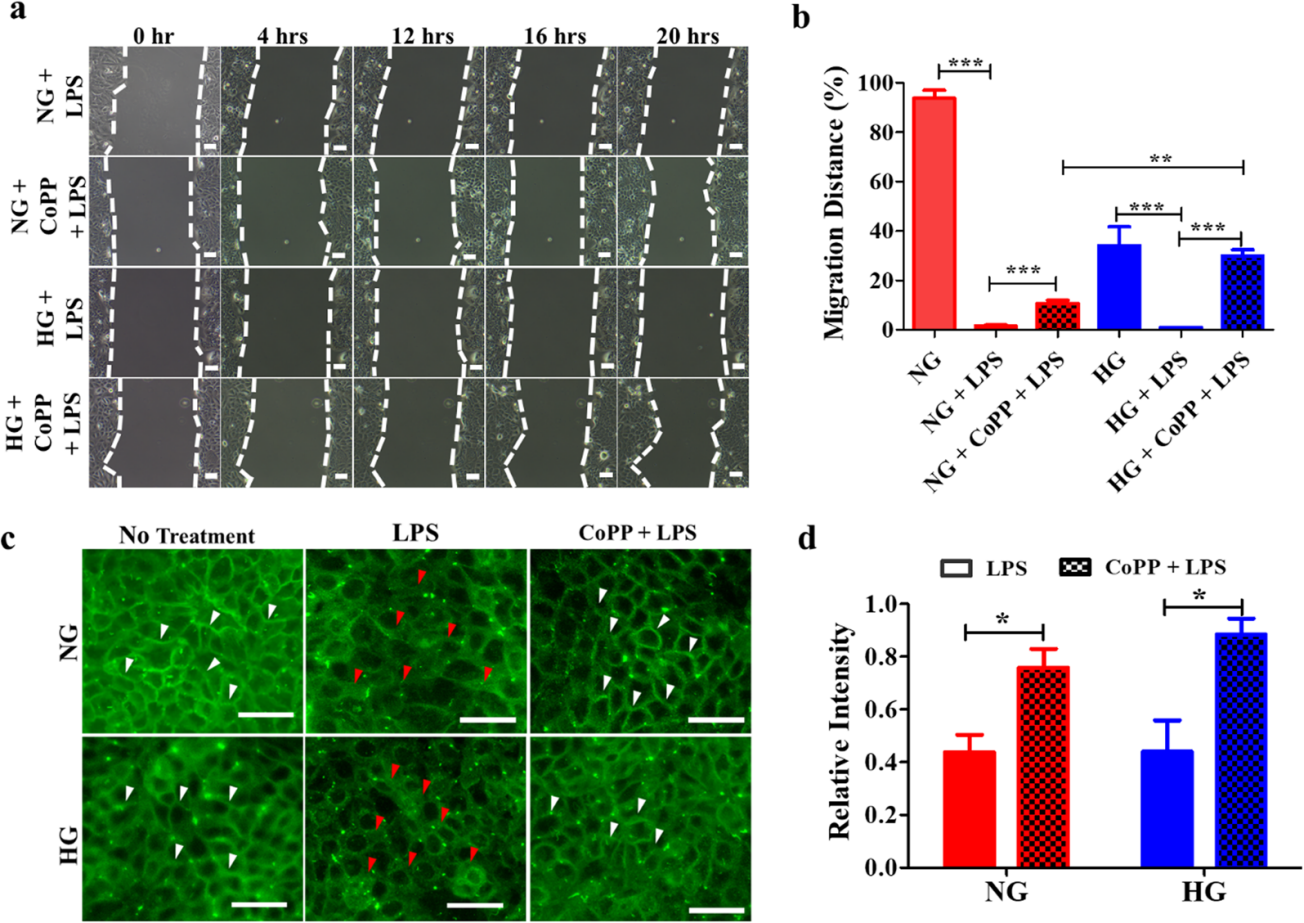
**T**he protective effect of CoPP pretreatment in HaCaT after exposing to LPS-containing media. **a** Time lapse images showing the migrated distance of CoPP-pretreated HaCaT after exposing to LPS-containing media (scale bar, 100 μm), **b** and the statistical analysis of the migrated distance of each sample at 20th hours of the migration assay. **c** The fluorescence probing of ZO-1 expressions in HaCaT that received LPS or CoPP pretreatment plus LPS (scale bar, 50 μm). **d** Statistic analysis of the fluorescence signals of ZO-1 in HaCaT that received either LPS or CoPP + LPS. The measured fluorescent intensities were normalized against the respective non-treatment group. **P < 0.01; ***P < 0.001

Apart from oxidative damage occurring with diabetic wounds, bacterial infection is another major cause that hinders the re-epithelization process (He et al. 2019). Accordingly, it was decided to investigate whether CoPP pretreatment could enhance the motility of HaCaT while culturing the cells in HG media containing lipopolysaacharide (LPS). It was noticed that in the presence of 1 µg/mL of LPS, HaCaT displayed minimal migration activity when compared to the untreated samples (Additional file 1: Fig. S7a), regardless of whether the cells were cultured in NG or HG media (Fig. 6a). The same concentration of LPS did not induce any cell death (Additional file 1: Fig. S7b**)**. Interestingly, the CoPP pretreatment significantly enhanced the motility of HaCaT in both media (Fig. 6b), indicating the pretreatment could promote keratinocyte migration even in the presence of LPS. Furthermore, CoPP was also demonstrated to enhance tight junction formation in HaCaT that were exposed to either LPS-containing NG or HG media **(**Fig. 6c). Since LPS is known to disrupt tight junction formation in keratinocytes, the protective effect of CoPP in tight junction formation after the addition of LPS to either NG or HG media was investigated. Zonula occludens-1 (ZO-1) is a well-known tight junction protein that is critical for tight junction formation in keratinocytes. Under normoglycemia, ZO-1 were clearly localized at the surface of HaCaT (white arrows), representing the tight junctions formed between the cells. In contrast, the fluorescence signals of ZO-1 detected in HaCaT cultured in HG media were fuzzy and less intense, suggesting hyperglycemia could downregulate tight junction formation in keratinocytes. Addition of LPS further downregulated the overall fluorescence intensity of ZO-1 detections in both media (red arrow), demonstrating LPS could disrupt tight junction formation regardless of the glucose concentrations. Conversely, the pretreatment of CoPP significantly upregulated ZO-1 expression (Fig. 6d), and the expressions were mostly localized at the cell membrane, suggesting the CoPP could maintain tight junction formation even in LPS-containing HG media.

## Discussion

Clinically, re-epithelization has been considered to be the most critical phase in diabetic wound healing. Hence recent studies on the development of novel therapies on diabetic wound healing have mainly focused on their capability to initiate re-epithelization in diabetic wounds (Rousselle et al. 2019). Likewise, the evaluation of the therapeutic benefit of CoPP in the present study was solely focused on its ability to initiate human keratinocyte migration under hyperglycemic conditions while in the presence of either H_2_O_2_ or LPS. Although, for clinical application, CoPP alone may not be sufficient to promote the entire healing process in a diabetic wound, it has been demonstrated to provide a synergistic effect when used in combination with stem cells. Recent in vivo studies have revealed CoPP could provide cytoprotection to stem cells that were intramyocardially-injected into the ischemic heart(Yao et al. 2018), as well as enhance the anti-apoptotic effect of stem cell therapy in an acute lung injury model (El-Shahat et al. 2021). Since stem cell therapy has been demonstrated to be effective in treating diabetic wounds, it is believed the findings in the present study will not just open up the possibility of clinical application of CoPP in the future for treating mild diabetic wounds, but also the possibility of establishing a combinatorial therapy of stem cells and CoPP in treating severe diabetic wounds.

Even though high glucose could induce cell apoptosis by upregulating the expression of cleaved caspase-3 (Jung et al. 2017), the increased glucose concentration could also induce increased concentration of phosphorylated Akt (p-Akt) in human keratinocytes (Rizwan et al. 2020). The increased presence of p-Akt could phosphorylate caspase-9 which in turn, downregulated the activation of caspase-3 and thus minimized cell death. Thus, this may partly explain why high glucose did not induce significant cell death as seen in the study of HaCaT proliferation, as the induced expression of p-Akt may have alleviated some of the hyperglycemia-induced damage in HaCaT. Conversely, hyperglycemia is known to suppress keratinocyte migration through the inhibition of the p38 MAPK pathway, which limited the migration capability of HaCaT. Accordingly, the data in the present study indicated high glucose had a limited effect on the proliferation of HaCaT, but could significantly reduce the migratory capability of the cells.

Interestingly, CoPP treatment was showed to limit cell proliferation in NG media, but not in HG media, after 5 days exposure. This was unexpected, since in the absence of any other stimulus the additions of CoPP are known not to have any effect on cell proliferation; although CoPP had recently been demonstrated to delay tumor growth (Barikbin et al. 2018). CoPP treatment also significantly decreased the migration rate of HaCaT cultured in NG media, as opposed to when the cells were cultured in HG media, in which the CoPP had significantly enhanced migratory capacity. It is well-established that CoPP can provide protection in oxidative injury through the induction of the Nrf-2/HO-1 pathway (Li et al. 2019). Additionally, HO-1 has been demonstrated to promote migration and transmigration in some cancer cells (Lu et al. 2012), although some studies have also indicated overexpression of HO-1 could downregulate the migratory capacity of some cell types (Abdelbaset-Ismail et al. 2017; Rodriguez et al. 2010). In the present study, however, the migratory capacity of CoPP-treated HaCaT was found to be influenced by the glucose concentration in the media. It is not known whether or how glucose metabolism could induce potential off-targeting of CoPP in HaCaT, as this would be a subject of interest for further study in the future.

Unexpectedly, the difference in the expression level of FoxO1 was clearly seen in the CoPP-treated HaCaT migrated in NG media when compared to those migrated in HG media. Originally, CoPP was considered to only induce HO-1 expression through the activation of Nrf-2; however, recently several studies have indicated that CoPP could also induce HO-1 expression through FoxO1 (Liu et al. 2013). Notably, the function of FoxO1 is controversial, and is likely influenced by the glucose concentration in the tissue microenvironment. Although, the overall expression level of FoxO1 in keratinocytes cultured under normoglycemic conditions was found to be similar to those cultured under hyperglycemic conditions, its function has been demonstrated to be remarkably different (Zhang et al. 2015). It was discovered that under hyperglycemic conditions, Foxo1 failed to bind to the *TGF*β1 promoter or stimulate *TGF*β1 transcription (Zhang et al. 2015). Instead, in high glucose, FoxO1 enhanced expression of CCL20 which is known to inhibit keratinocyte migration (Furue et al. 2020). However, some studies have also demonstrated that a high level of FoxO1 could inhibit cell migration even under normoglycemic conditions (Pan et al. 2017). In the present study, the FoxO1 expression in CoPP-treated HaCaT was significantly higher in NG media compared to HG media, and that the higher expression correlated with high expression of CCL20 and low expression of TGFβ1, which could explain why the CoPP-treated cells migrated significantly more slowly in NG than in HG media.

The fact that CoPP enhanced keratinocyte migration in high glucose, as opposed to the inhibition of the cell migration and proliferation in normal glucose, actually justifies its future application in diabetic wound treatment. Clinical studies have revealed that several therapeutic agents that promote diabetic wound healing, could also increase the chances of diabetic patients developing cancers (Sundaram et al. 2018). It was found that in addition to inducing apoptosis in keratinocytes, hyperglycemia was found to restrain cutaneous epithelial plasticity, which was necessary for functional wound closure (Tan et al. 2021). This may explain why excessive therapeutic agents are often needed to achieve successful wound closure in diabetic wounds. However, it was found these agents could also be induced in epithelial cells to undergo epithelial–mesenchymal transition under normoglycemic conditions which ultimately led to cancer development (Sundaram et al. 2018). Thus, from a clinical perspective, CoPP could be an excellent healing agent for treating diabetic wounds during the mid or late phase of the healing process, since protoporphyrin could continue to sustain keratinocyte migration under hyperglycemic conditions while minimizing uncontrollable keratinocyte proliferation and migration under normoglycemic conditions.

Interestingly, when exposed to either H_2_O_2_ containing NG or HG media, the strong expression of FoxO1 seemed to favor strong migratory capability of CoPP-pretreated HaCaT in both media. Although, the pro-survival effect in CoPP-pretreated HaCaT resulted in the enhancement of migratory capability because of the induction of the Nrf-2/HO-1 pathway, we believe that the CoPP-induced FoxO1 may have also played a role. It has been noted that when mitochondria suffered from cellular damage due to the presence of H_2_O_2_ or high glucose, FoxO1 could translocate to the mitochondria and activate the expression of PGC-1α, a transcriptional coactivator that is known to promote mitochondrial biogenesis (Wu et al. 2015). Accordingly, the cytoprotection of HaCaT exerted by CoPP while in the presence of H_2_O_2_ was likely the synergistic effect of the induced expressions of Nrf-2/HO-1 and FoxO1. Obviously, future study is warranted to study the relationship between CoPP treatment and mitochondrial biogenesis in the presence of high glucose and H_2_O_2_.

Similar to the H_2_O_2_-induced oxidative damage, LPS-induced cellular damage in HaCaT could be alleviated through activation of Nrf-2/HO-1 pathway (He et al. 2019). It is well-established that LPS treatment can also inhibit keratinocyte migration and disrupt the tight junction formation (Pfalzgraff et al. 2016). Indeed, the CoPP-pretreatment significantly increased the migratory capability of the HaCaT while in either LPS-containing NG or HG media. Moreover, the localization of ZO-1 at the tight junction was also significantly elevated in the HaCaT that had received CoPP pretreatment. This indicated that CoPP-pretreatment could potentially provide protection against bacterial infection that occurred during diabetic wound healing.

## Conclusions

**Fig. 7:**
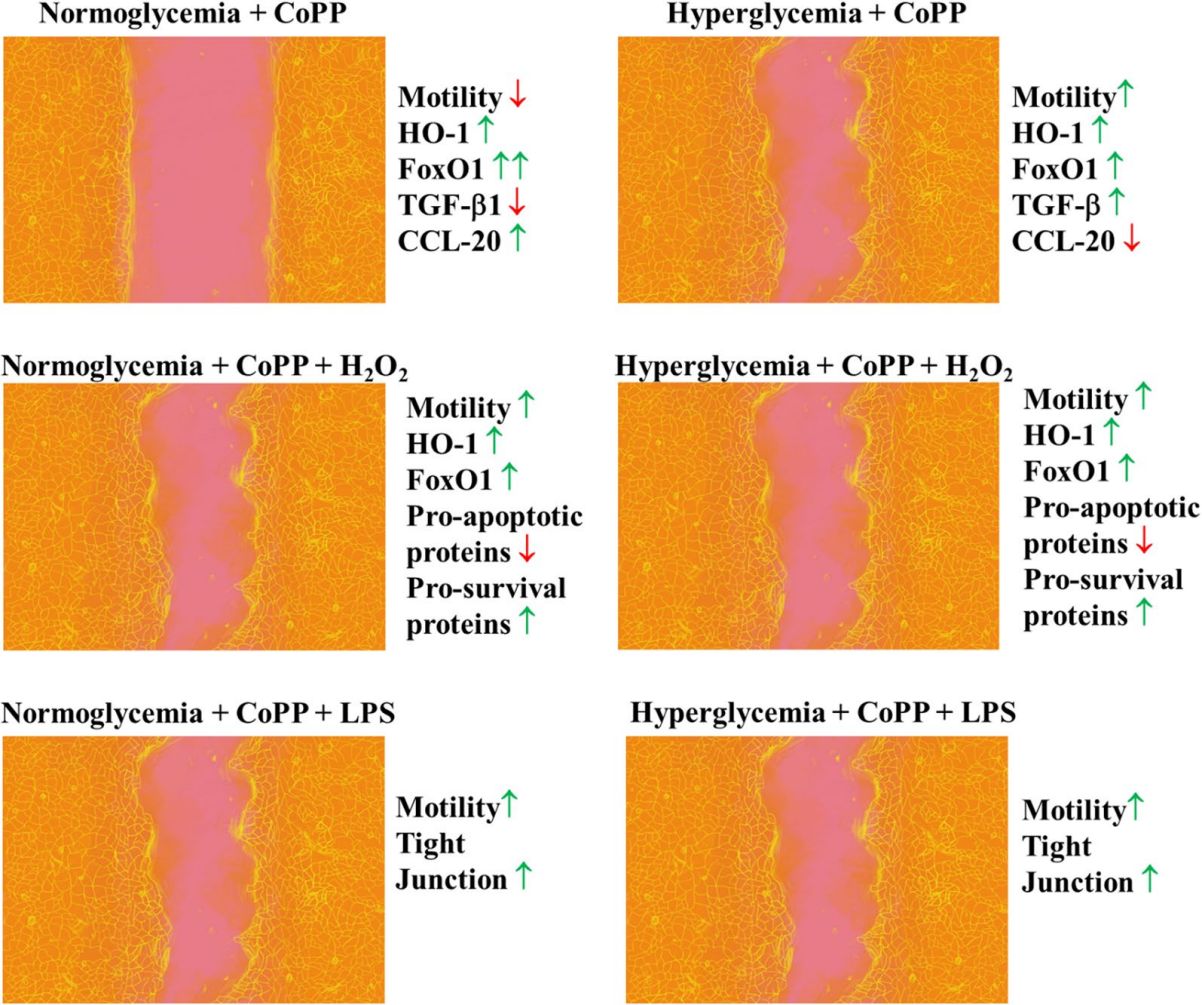
Schematic diagram showing the summary of therapeutic effect of CoPP in promoting HaCaT migration. CoPP could downregulate HaCaT’s migratory capability when the cells were cultured in NG media. In contrast, compared to untreated samples, CoPP-treatment had significantly enhanced the HaCaT’s migratory capability when being cultured in HG media. Likewise, in the presence of either H_2_O_2_ or LPS, the motility of HaCaT were increased when being pretreated with CoPP

In this study, CoPP was demonstrated to promote keratinocyte migration in an in vitro wound healing assay under hyperglycemic conditions (Fig. 7), and that CoPP pretreatment was found to downregulate the expression level of pro-apoptotic proteins when the cells were cultured in either glucose condition in presence of H_2_O_2_. It is believed CoPP has the potential to become a healing agent for treating diabetic wounds, especially in conjunction with robust healing agents such as growth factors and stem cells.

## Supplementary Information

**Additional file  1.  Fig. S1.** Schematic diagram showing the live-cell imaging protocol of CoPP treated HaCaT underwent migration in either normal glucose or high glucose media. **Fig. S2.** The influence of CoPP on the motility of WS1 in in vitro wound healing assay.** Fig. S3.** Quantitative analysis of the protein expressions in CoPP-pretreated HaCaT cultured in either NG or HG media. **Fig. S4.** Quantitative analysis of the protein expressions in CoPP-treated WS1 cultured in either NG or HG media. **Fig. S5. **Viability of CoPP-pretreated HaCaT cultured in either normal glucose or high glucose in the presence of 300 µM H2O2. **Fig. S6** Schematic diagram showing the live-cell imaging protocol of CoPP pretreated HaCaT cultured in either low glucose or high glucose after H2O2 exposure. **Fig. S7.** Viability of HaCaT cultured in LPS-containing media

## Data Availability

All materials are available by the corresponding authors.
