# Supplementary material for: Cobalt protoporphyrin promotes human keratinocyte migration under hyperglycemic conditions
Source: Mol Med. 2022 Jun 23;28:71. doi: 10.1186/s10020-022-00499-0 (PMC9219158; doi:10.1186/s10020-022-00499-0)
Supplement: Supplementary file 1 — Additional file 1. Fig. S1. Schematic diagram showing the live-cell imaging protocol of CoPP treated HaCaT underwent migration in either normal glucose or high glucose media. Fig. S2. The influence of CoPP on the motility of WS1 in in vitro wound healing assay. Fig. S3. Quantitative analysis of the protein expressions in CoPP-pretreated HaCaT cultured in either NG or HG media. Fig. S4. Quantitative analysis of the protein expressions in CoPP-treated WS1 cultured in either NG or HG media. Fig. S5. Viability of CoPP-pretreated HaCaT cultured in either normal glucose or high glucose in the presence of 300 µM H2O2. Fig. S6 Schematic diagram showing the live-cell imaging protocol of CoPP pretreated HaCaT cultured in either low glucose or high glucose after H2O2 exposure. Fig. S7. Viability of HaCaT cultured in LPS-containing media [file 10020_2022_499_MOESM1_ESM.docx]

**Cobalt Protoporphyrin Promotes Human Keratinocyte Migration under Hyperglycemic Conditions**

Peng- Hsiang Fang^1^, Ying-Ying Lai^2^, Chih-Ling Chen^2^, Hsin-Yu Wang^2^, Ya-Ning Chang^3^, Yung-Chang Lin^1^, Yu-Ting Yan^4^, Cheng-Hung Lai^1*^, Bill Cheng^3*^

**Affiliation**

^1^Department of Veterinary Medicine, National Chung-Hsing University, Taiwan

^2^Bachelor Program of Biotechnology, National Chung-Hsing University, Taiwan

^3^Graduate Institute of Biomedical Engineering, National Chung-Hsing University Taiwan

^4^Institute of Biomedical Science, Academia Sinica, Taiwan

**Correspondence**

Bill Cheng, Ph.D.

Graduate Institute of Biomedical Engineering

National Chung-Hsing University

No.145, Xing Da Road, Taichung, 402, Taiwan

Phone: +886-4-22840165 #936

Email: [bcheng@dragon.nchu.edu.tw](mailto:bcheng@dragon.nchu.edu.tw)

Cheng-Hung Lai, PhD

Department of Veterinary Medicine

National Chung-Hsing University

No.145, Xing Da Road, Taichung, 402, Taiwan

Phone: +886-4-22870180 #204

Email: [chlai@dragon.nchu.edu.tw](mailto:chlai@dragon.nchu.edu.tw)

**Supplementary Figures**
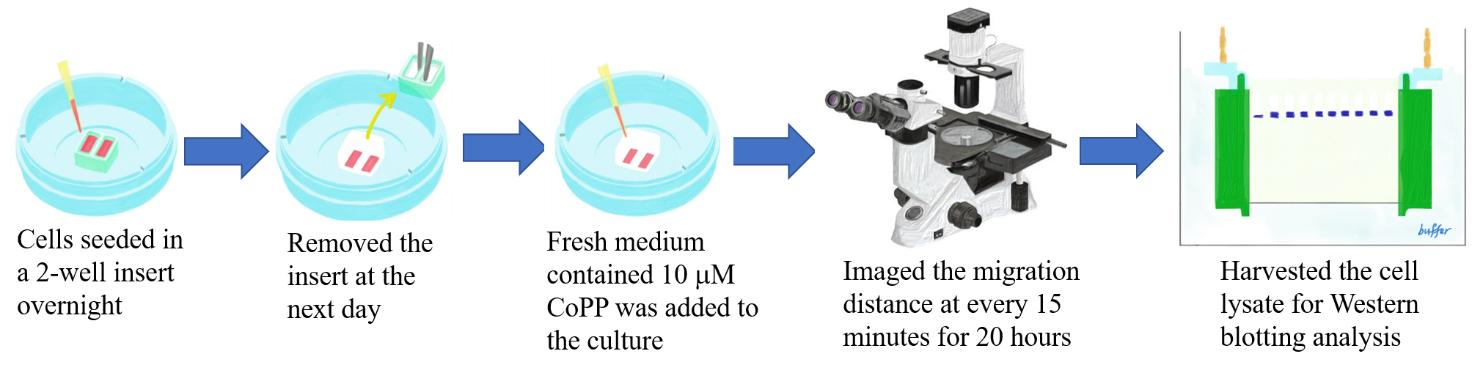


**Fig. S1. Schematic diagram showing the live-cell imaging protocol of CoPP treated HaCaT underwent migration in either normal glucose or high glucose media.**


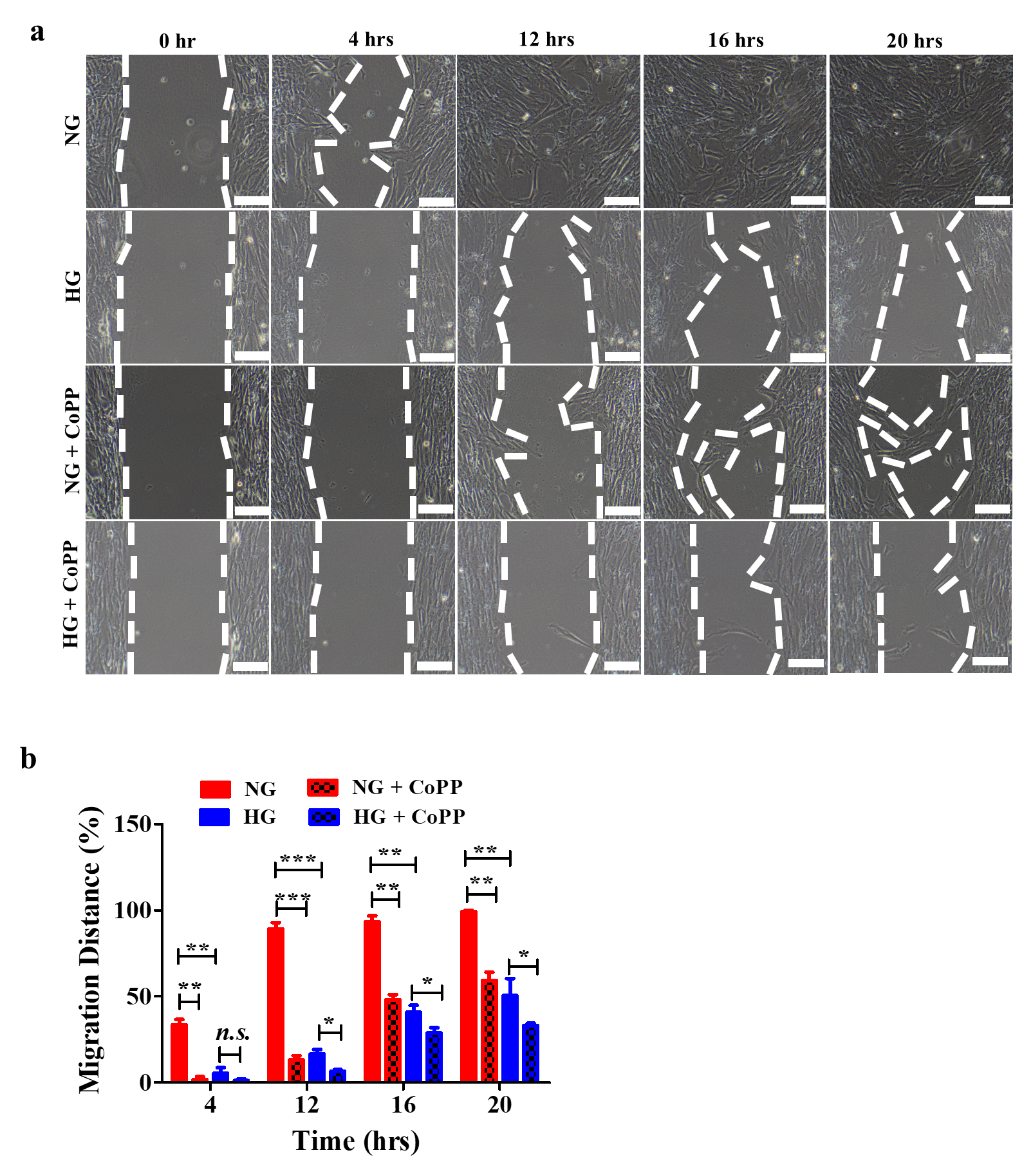


**Fig. S2. The influence of CoPP on the motility of WS1 in *in vitro* wound healing assay.** (**a**) Time lapse images showing the migrated distance of WS1 after exposing to CoPP containing NG or HG media (scale bar, 100 μm), (**b**) and the statistical analysis. *, *P* < 0.05; **, *P* < 0.01; ***, *P* < 0.001.


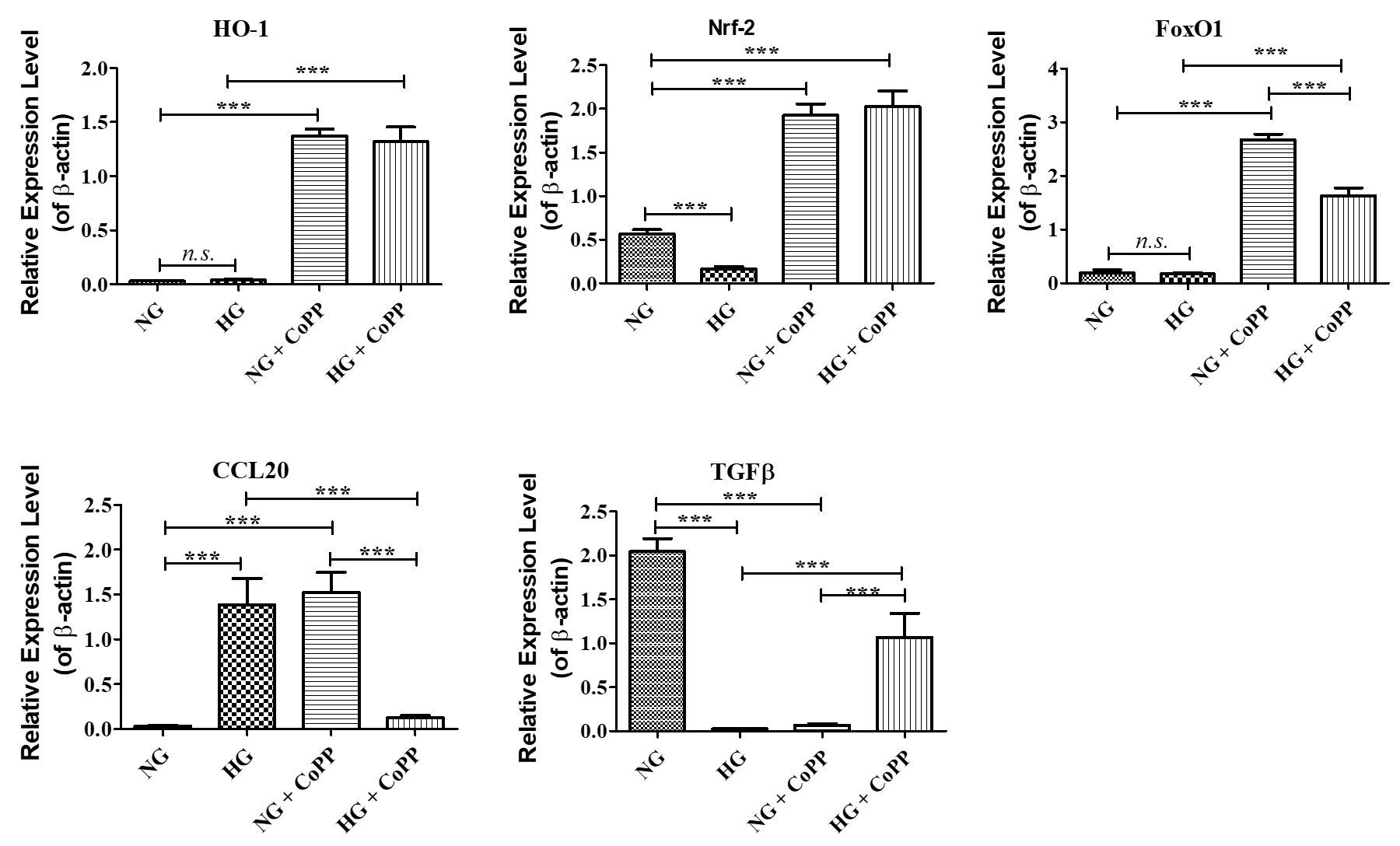


**Fig. S3.** **Quantitative analysis of the protein expressions in CoPP-pretreated HaCaT cultured in either NG or HG media.** HaCaT were cultured in either NG or HG media and exposed to 10 μM CoPP for 24 hours. Cell lysates were harvested and subjected to Western blotting analysis. The expression level of each protein marker in all the samples were quantified and analyzed (N=3). *, *P* < 0.05; **, *P* < 0.01; ***, *P* < 0.001; *n.s.*, not significant.


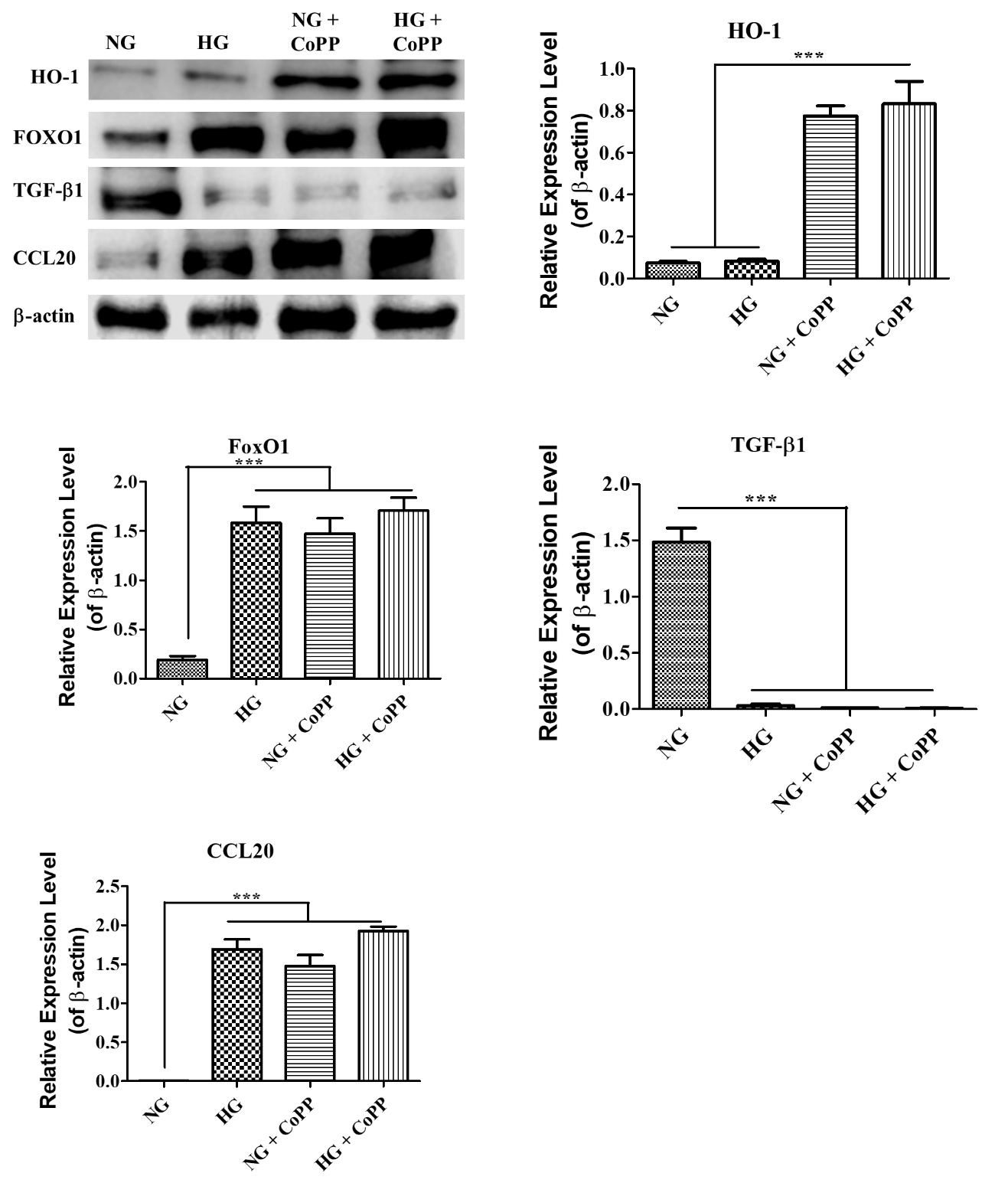


**Fig. S4.** **Quantitative analysis of the protein expressions in CoPP-treated WS1 cultured in either NG or HG media.** WS1 underwent migration in either NG or HG media while exposing to 10 μM CoPP for 20 hours. Cell lysates were harvested and subjected to Western blotting analysis. The expression level of each protein marker in all the samples were quantified and analyzed (N=3). ***, *P* < 0.001.


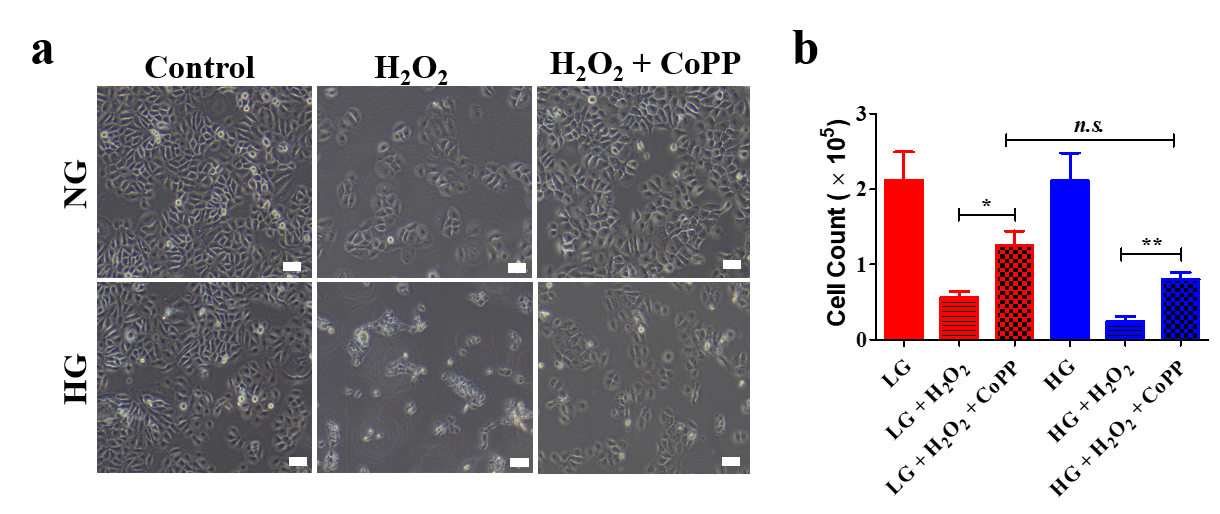


**Fig. S5. Viability of CoPP-pretreated HaCaT cultured in either normal glucose or high glucose in the presence of** **300 μM H_2_O_2_.** (a) Images of CoPP pretreated HaCaT after exposed to 300 μM H_2_O_2_ overnight in either low glucose or high glucose (scale bar, 10 μm), and the statistical analysis (*, *P* < 0.05; **, *P* < 0.01; *n.s.*, not significant).


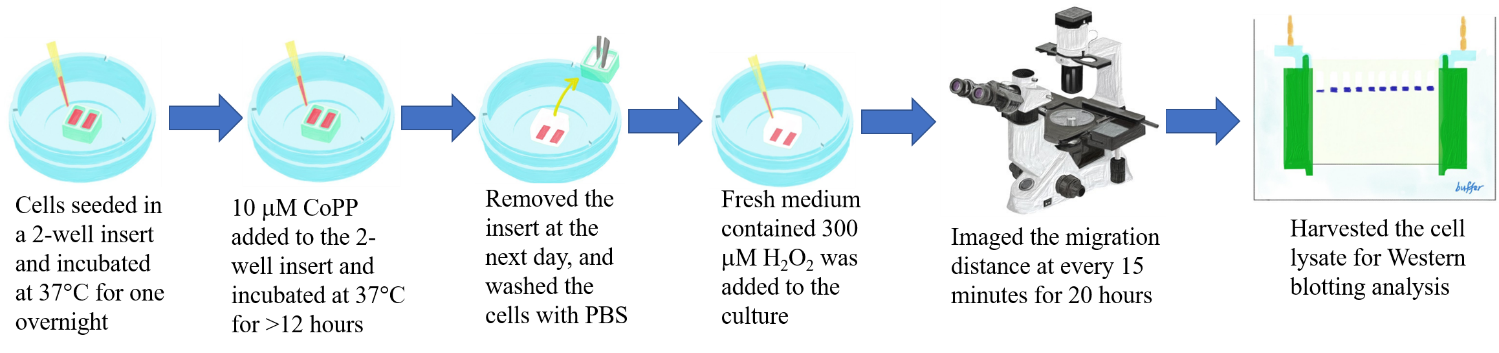


**Fig. S6 Schematic diagram showing the live-cell imaging protocol of CoPP pretreated HaCaT cultured in either low glucose or high glucose after H_2_O_2_ exposure.**


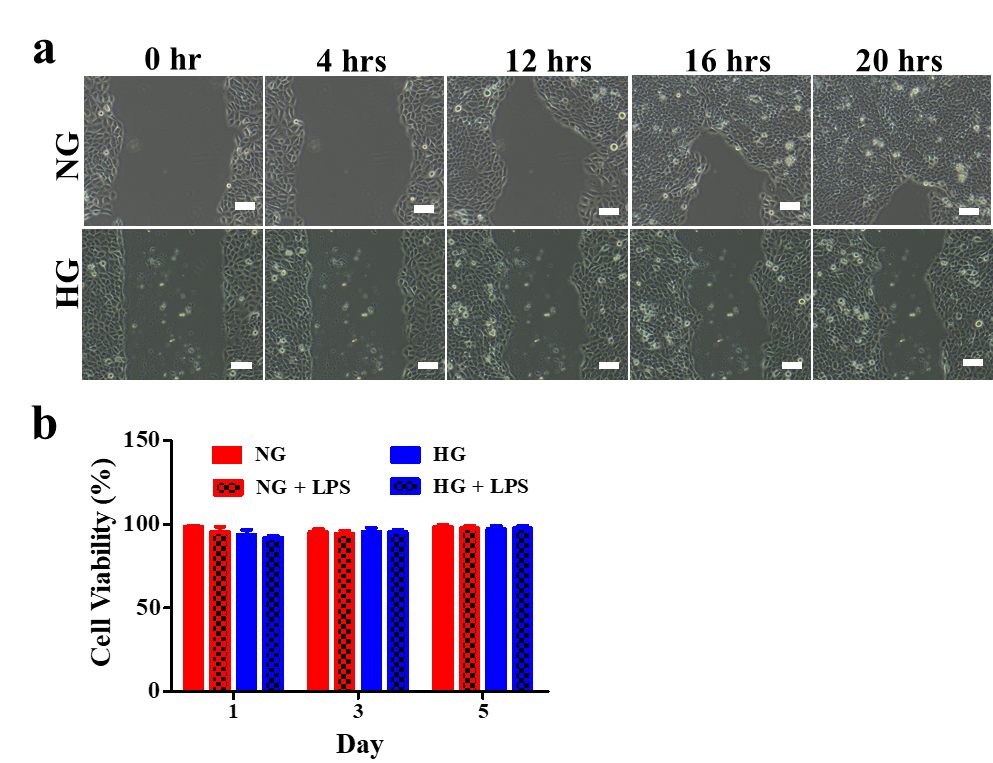


**Fig. S7. Viability of HaCaT cultured in LPS-containing media.** (**a**) Representative images of untreated HaCaT underwent cell migration in either NG or HG media for 20 hours (scale bar, 10 μm). (**b**) The cell viability of HaCaT exposed to 1 μg/mL LPS for 5 days while cultured in either NG or HG media.
